# Interstitial lung disease associated to erlotinib treatment: a case report

**DOI:** 10.1186/1757-1626-3-59

**Published:** 2010-02-12

**Authors:** Yolanda del Castillo, Paulina Espinosa, Fernanda Bodí, Raquel Alcega, Emma Muñoz, Carlos Rabassó, David Castander

**Affiliations:** 1 Unidad de Cuidados Intensivos, Hospital de Sant Pau i Santa Tecla, Rambla Vella, 14. 43003, Tarragona, Spain

## Abstract

**Introduction:**

Few cases of pulmonary toxicity related to epidermal growth factor receptor-targeted agents have been described.

**Case presentation:**

We report a case of a 63-year-old white male with stage IV non-small cell lung cancer treated with erlotinib who developed a interstitial lung disease.

**Conclusion:**

Respiratory symptoms during treatment with erlotinib should alert clinicians to rule out pulmonary toxicity. Early erlotinib withdrawal and corticoid administration were successful.

## Introduction

The human epidermal growth factor receptor (EGFR) is a transmembrane glycoprotein that consists of an extracellular ligand-binding domain, a hydrophobic transmembrane region, and an intracellular tyrosine kinase domain [1]. The union with the ligand-binding activates tyrosine-kinase and initiates a signalling cascade that leads to cell proliferation, increased angiogenesis, metastasis and decreased apoptosis [2]. Over-expression of EGFR has been reported in a wide variety of solid tumour types: lung, pancreatic, head and neck, colorectal, breast, renal, glioma, prostate, ovarian and bladder [1]. Epidermal growth factor receptor-targeted agents have been investigated as second- and third-line treatment [3]. The most developed agents in clinical research are the intracellular EGFR tyrosine kinase inhibitors (EGFR TKIs) (gefitinib, erlotinib) and the monoclonal antibody aimed at extracellular ligand-binding domain (cetuximab) [2]. Gefitinib (ZD1839, Iressa^®^; AstraZeneca) has been approved for non-small cell lung cancer (NSCLC), and erlotinib (OSI-774, CP-358,774, Tarceva^®^; OSI Pharmaceuticals in collaboration with Genentech and Roche Pharmaceuticals) has also been approved for NSCLC and is in a Phase III trial in patients with pancreatic cancer. Adverse effects of gefitinib and erlotinib are rash, diarrhea, asthenia and anorexia. Interstitial lung disease (ILD) is an infrequent and usually fatal complication [4]. We present a case of severe ILD in a patient with NSCLC treated with erlotinib who survived.

## Case Presentation

A 63-year-old non-smoker male presented with non-productive cough and hypereosinophilia was finally diagnosed of stage IV NSCLC (T2aN2 M1) with visceral involvement affecting contralateral lung, liver and brain. Paraneoplastic hypereosinophilia was resolved with prednisone. Cisplatin and vinorelbine were given during one month but needed withdrawal because of transient acute renal insufficiency related to cisplatin. A second line treatment with erlotinib (150 mg daily) in addition to prednisone was initiated. Dramatic improvement of symptoms and radiographic regression were induced.

**Figure 1:**
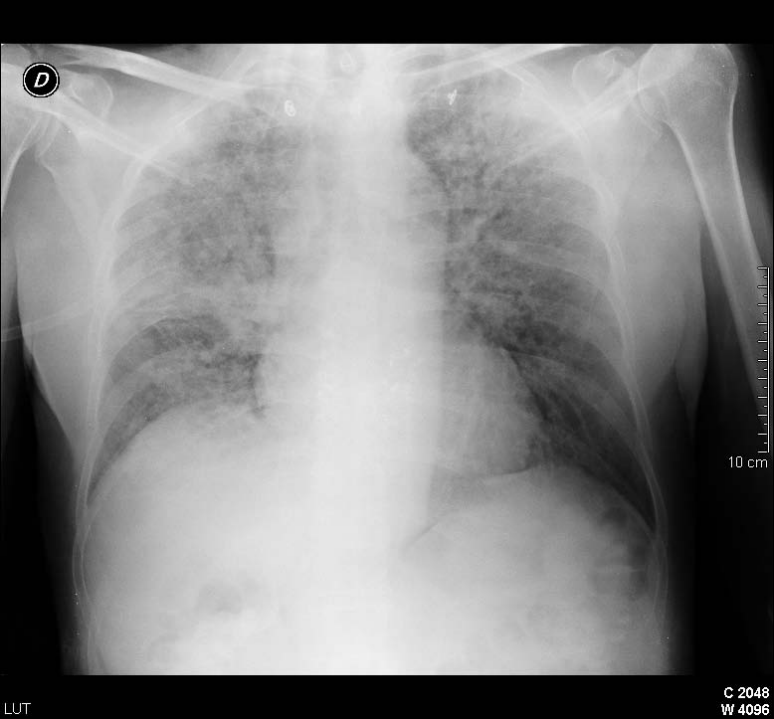
**Diffuse bilateral interstitial infiltrates were predominant in upper lobes**. Right hiliar consolidation corresponds to the tumor.

**Figure 2:**
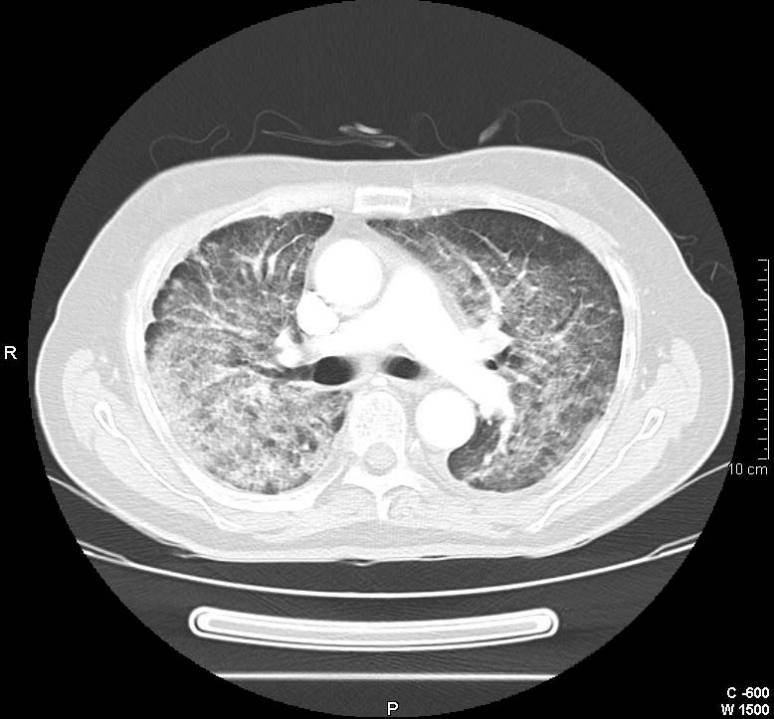
**Pulmonary CT scan shows extensive ground glass opacities**.

After seven weeks of treatment fever and chills appeared without demonstrable infection, coinciding with reduction of the steroid dose from 20 mg to 1 mg/day of prednisone. General conditions deteriorated in the following seven days, and dyspnea, pulmonary infiltrates and progressive respiratory failure developed (fig. 1). Computed Tomography showed increased interstitial parenchyma density affecting both lungs (fig. 2). Heart failure was also excluded: an echocardiography reported normal ventricular chambers without dilatation nor hypertrophia, no contractility impairment with left ventricular ejection fraction 74%, normal atrial chambers and no valve abnormalities. Erlotinib treatment was immediately discontinued and methylprednisolone (1 mg/kg daily) was initiated. Empirical treatment with imipenem, clarithromycin, trimethoprim - sulfamethoxazole, amphotericin B and ganciclovir were given. Patient was admitted in the Intensive Care Unit.

Despite therapy, the patient developed an acute lung injury in accordance with the Lung Injury Score definitions [5], and required mechanical ventilation with FiO2 0.6, tidal volume 450 ml, PEEP 10. Peak airway pressure was 25 cm H2O and PaO2/FiO2 225. Cardiovascular evaluation ruled out heart failure. Flexible fiberoptic bronchofibroscopy revealed minimal inflammatory changes. Bronchoalveolar lavage, protected catheter brush and bronchial specimen cultures excluded acid-fast resistant bacilli, bacterial or fungal infections. Eosinophilic pneumonitis and malignancy were also discarded. Polymerase chain reaction analysis (PCR) for *Herpesviridae *and *Mycobacterium tuberculosis *were performed in blood and bronchial samples, proved also negative. The Papanicolau stain in bronchoalveolar lavage samples, and the hematoxylin-eosin stain in transbronchial biospy samples discarded *Pneumocystis jiroveci *infection. Blood and urine cultures were negative. Urinary antigen testing for *Legionella *and *Pneumococcus *were also negative. Consequently, antibiotic treatment was discontinued at day five.

**Figure 3:**
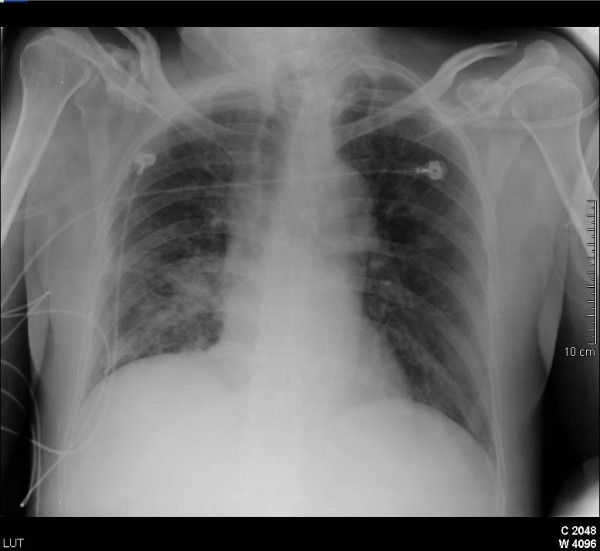
**(Six days later) important reduction of the bilateral infiltrates; previous consolidation is still present**.

**Figure 4:**
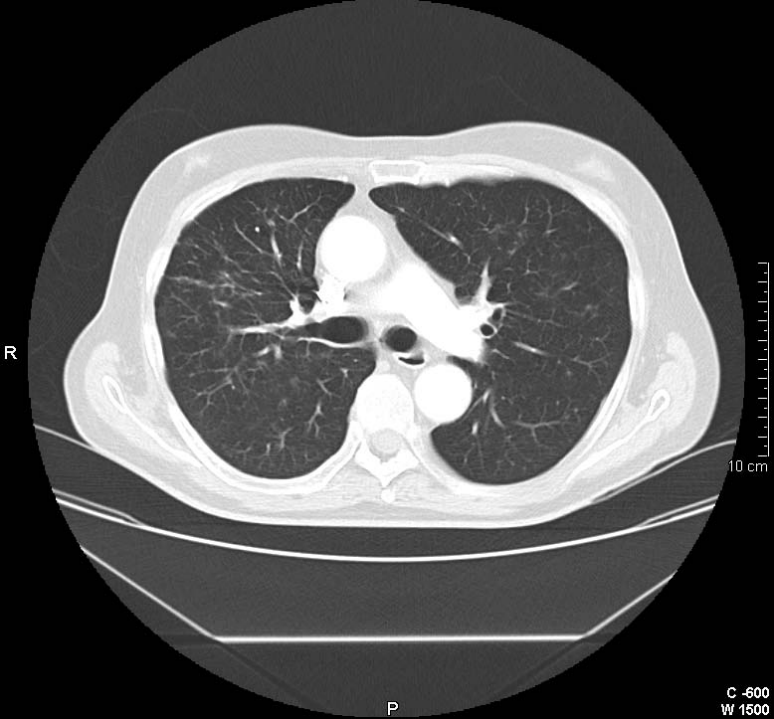
**(Three months later) a CT scan shows almost complete resolution with minimal residual reticular opacities in the right lung**.

On the following days gas-exchange improved and pulmonary infiltrates progressively solved (fig. 3). It was possible to wean off the patient from the ventilator and was successfully extubated on the fourth day of intensive care. On the sixth day he was discharged from the ICU to a normal ward with the diagnosis of interstitial pneumonitis induced by erlotinib. Three months later a CT scan was performed showing no abnormalities (fig. 4).

## Discussion

ILD associated with gefitinib use has been reported in approximately 1% of patients worldwide, with worse evolution in current or former smokers and pre-existing pulmonary fibrosis [4,6]. There are few cases reported of lung toxicity related to erlotinib use. A phase III trial TRIBUTE, a randomized placebo-controlled trial, examined the efficacy and safety of erlotinib versus placebo in combination with paclitaxel and carboplatin for first-line therapy of advanced NSCLC in 1,079 patients. There were five severe ILD-like events in the erlotinib arm (1.0%), versus one event in the placebo arm (0.2%). All ILD-like events were fatal [7]. There are two cases reported of ILD induced by erlotinib treatment, one of them a 60-year-old patient former smoker with previous pulmonary fibrosis [4], the other one a 55-year-old current smoker with chronic obstructive lung disease [8]. Both of them died. After a review of all cases already published, we report the case of a patient that survived after an episode of severe ILD related to erlotinib.

Diagnosis of pulmonary toxicity should be made early because of its potential increased mortality. High suspicion is necessary in those patients under treatment with erlotinib who develop respiratory symptoms like dyspnea, cough or fever. It is an exclusion diagnosis beyond other possible causes like congestive heart failure, infection or lymphangitic carcinomatosis [8]. In our patient, erlotinib withdrawal and corticotherapy (metilprednisolone 1 mg/kg/day) were successful.

The etiology of ILD related to EGFR-tyrosine kinase inhibitor therapy is poorly understood. Based on case reports, common histopathologic evaluations have revealed diffuse alveolar damage with hyaline membrane formation. The molecular mechanisms leading to pulmonary toxicity are also unclear [4].

## Conclusion

In conclusion, clinicians should have a high suspicion for the diagnosis of pulmonary toxicity when respiratory symptoms appear in a patient while receiving erlotinib treatment. Early withdrawal of erlotinib and treatment with corticoids may improve prognosis. Further investigation is necessary to establish if concomitant use of corticoids and erlotinib can lower the incidence of pulmonary toxicity associated to the EGFR-inhibitors treatment. Research is necessary to determine the physiopathology and predisposing factors in the development of pulmonary toxicity related to erlotinib.

## Abbreviations

EGFR: epidermal growth factor receptor; EGFR: tyrosine kinase inhibitors; EGFR: TKIs; ILD: interstitial lung disease; NSCLC: non-small cell lung cancer; PCR: polymerase chain reaction; TKI: tyrosine kinase inhibitor.

## Consent

Written informed consent was obtained from the patient for publication of this case report and accompanying images. A copy of the written consent is available for review by Editor-in-Chief of this journal.

## Competing interests

The authors declare that they have no competing interests.

## Authors' contributions

YC, PE, FB, RA, EM, CR and DC participated in the medical interventions. YC and PE were the major contributor in writing the manuscript. All authors read and approved the final manuscript.
